# Odontological Society

**Published:** 1858-01

**Authors:** 


### ARTICLE XX.

### *Odontological Society.*

NOV'R 2, 1857.—Arnold Rogers, Esq., V. P., in the chair.

This being the first meeting of the session 1857–'58, the chairman made a few introductory remarks on taking the chair. He expressed his satisfaction at seeing so many members present, and congratulated them that mortality had not visited the ranks of the society during the past session. He said that they had still, however, to regret the absence of their worthy president, whose health continued in such a state as to preclude the possibility of his taking the chair; as also, Mr. Nasmyth, a member of the council, who was prevented from attending their meetings from a similar cause. He trusted that the future meetings of the coming session would be as numerously attended as the present; and be conducted with the same harmony, and be productive of the same good results, as those of the last session.

Mr. Alexander Marshall Duff then read a paper "On the Materials used by Dentists." The author commenced by stating that he was desirous of being understood as having no wish to lay down laws to be obeyed or rules to be followed, as in the selection of the materials best fitted, every dentist, he had no doubt, was actuated by a desire to do the best he could, but he was desirous of giving a somewhat lengthened testimony to the materials he had found to be the most suitable and durable. And without going scientifically into the component parts or chemical constituents of the materials used by dentists, he thought it would best answer the purpose he had in hand, to speak of that only of which he had a personal knowledge. Mr. Duff said it would be inconsistent with fact, were he to speak of the dentists as a *body* some twenty-five years ago, but he thought that there was a sufficient approximation of practice among the leading dentists in this country at that time, to warrant him in saying, that the materials then in use were metallic and animal, with the occasional use of a mineral substance. The metallic substances were gold, silver and platina, but the latter being difficult to obtain commercially, was used in small quantities, and that principally in form of wire. He considered gold, then, as now holding the first place among the metals, owing to its extreme applicability to dental purposes, and because of its hardness, ductility, purity, and unoxydizable character in the mouth. He proceeded to compare silver and platina with gold, and said that gold would always hold the first place among the metals used. The author then alluded to the animal substances in use, and in naming them gave priority in value as they were classed: they were hippopotamus tusk, walrus tusk, whale teeth, and ivory, and with the exception of ivory, the same substances were still used, but he considered hippopotamus as being by far the most suitable of all the animal substances; in its density it more nearly resembled dentine: he pointed out several peculiarities possessed by walrus tusks, capable of a considerable resistance to the action of acids in the mouth, and admitting of a high degree of polish; still, from its softness, he considered it less valuable: and with respect to whale teeth, he was not in a position to speak of its lasting properties, but he had an objection to use it from its offensive odor and bad color; he considered it as the coarsest in grain, and commercially the cheapest of all. Natural teeth, he thought, might best be considered as dental substitutes, comparatively, with the mineral teeth of the present day. After enumerating the various combinations of these materials as dental structures, he said, that he had seen artificial work that in point of strength, nicety of adaptation and beauty of finish, if it did not surpass, has seldom been excelled by the skillful artificer of the present day, and paid a high compliment to the early practitioners in the profession, and thought it a duty incumbent on the practitioners of the present, not to lose any of the prestige belonging to the practice of dentistry. The author then alluded to mineral teeth, and considered their introduction into the profession a step in the right direction, and contrasted the minerals of French manufacture with those at present in use. He said, that it is now nineteen years since the late Mr. Claudius Ash first made a mineral tooth. Although they went far in advance of the French teeth, still had it not been for subsequent improvements, their adoption would have been rejected, as they were extremely brittle. Without contrasting the qualities of mineral teeth as made by various manufacturers of the present day, he considered that there was not a perfect mineral tooth to be had, but hoped, that ere long, various improvements would lead to this desirable end. He thought that if a tooth could be made combining the lustre of Lemale's, the strength of Taylor's, and the finish of Ash's, it would go far to obviate the evil spoken of. It was a matter of great importance to dentists, seeing that mineral teeth have now nearly superseded the use of naturals. The liability of mineral teeth to fracture, was a cause of great annoyance, which the exercise of skill could not do away with. Mineral teeth had also introduced the element of competition in the profession, and had been the stalking-horse of many an imposter; the words indestructible, incorrodible, had been freely used in their praise; the introduction of palladium, dental alloy, and other white metals, were alluded to, and were considered by the author as quite unfit for the purposes they were used. Their introduction might be justified, were they suitable to construct artificial for poor persons, but in his opinion, there was no suitable material, or combination of materials at present, proper to be used in the construction of artificial work for the poor. Gutta percha was next alluded to as a base on which to construct artificial work, but from its liability to decay, and decay accompanied with such offensiveness, in the opinion of the author it was deemed most objectionable. Having thus alluded to most if not all the materials used in combination for the construction of artificial work, Mr. Duff said that he had intentionally avoided naming the materials used in stopping teeth, from a conviction of the importance of the subject—a subject that seemed to be the dental problem of the day—when, with what, and how a tooth ought to be stopped; he trusted the time was not far distant when the council of this society may see fit to appoint a committee of its members, whose duty shall be to analyse and investigate all the materials now in use, and that may be recommended for that purpose. He felt assured that this society possessed gentlemen eminently qualified to discharge so important a duty as that of watching over the purity of our materials. Then we should have an authority to quote, a standard to refer to. In conclusion, he apologised for the discursiveness of the paper; he had endeavored to be as concise as possible.

The chairman, referring to Mr. Duff's observations on the harder form of platina, said it would be a disideratum for the poor if it could be generally adopted, but when he (the chairman) had worked with platina in former years, he had observed that it soon became brittle, which he thought was a great objection to its use. In regard to silver plates, many of which he had made for poor people in hospital practice, it was no uncommon thing to see them become black and dissolve away, from the acidity of the saliva, or some other cause. As to gutta percha, however beautifully manufactured, he had observed, from some peculiar action going on in the mouth under its use, that those parts of the teeth surrounded by the gutta percha became affected with a peculiar white decay. He had known instances in which persons, who had previously worn gold plates and gold clasps with *impunity*, on having changed them for gutta percha, had had their teeth so affected. He had that day stopped three teeth under those circumstances. This he thought arose from the gutta percha retaining small particles of food and the secretions of the mouth, more than gold did, thus producing decay upon the necks of the teeth.

With regard to bone, he had seen it three or four times in his life become perfectly black on its surface (as black as ebony, and almost as hard) under use in the mouth. In natural teeth, also, the same peculiarity was sometimes observed. He knew a gentleman whose teeth, when he was a boy about sixteen or seventeen years old, were affected with white decay, and who was told that all his teeth would become decayed before he was twenty. Gradually, however, the teeth assumed a brownish color, and before he was twenty they became black. He (the chairman) saw him with those teeth at the age of fifty-two, no further decay having taken place in them up to that age. In reference to servants, he thought it undesirable that they should be encourged to use artificial teeth to any great extent; and he generally advised masters and mistresses to abstain from presenting them (which they sometimes did) with entire sets of teeth, unless they intended to leave them a legacy to keep up the necessary repairs which they would require in after life.

Mr. Spence Bate said he had occasionally seen the same black appearance in the teeth as that alluded to by the chairman; and had sometimes remarked that they were black in one portion, hard, firm, and durable, while other parts of the same teeth were white, soft, decayed, and required filling. Speaking of artificial substitutes, he remembered a case in which a patient had worn a set of ivory blocks (according to her statement) eleven years. They become hard, and as yellow as brimstone; but were as perfect as when they were first put in. His experience differed from that of the author's, in regard to the durability of the hippopotamus tusk. It was the hardest bone used by dentists, but decayed, he thought, the most rapidly. It would take the highest polish, which was probably the cause of its more rapid decay. It contained more carbonate of lime than any other of the ivories. The consideration of this subject showed how absolutely necessary it was that the dentist should, as soon as possible, arrive at a distinct idea as to what acid it is that induces a destruction of the material. In this respect, his own opinion, he believed, differed from that of others. In his opinion, it would be found that carbonic acid was the agent of disintegration. He believed that carbonate of lime existed in dentine, not as an integral part, since phosphate of lime was the only salt found in the blood, and that consequently the carbonate must be formed during the process of development of dentine, and that it existed in an amorphous state more or less in the tubuli. These remarks he hastily threw out, not as a perfected theory, but for the consideration of the members. He had had very little experience in the use of gutta percha. When it was first introduced, he tried it as an experiment on a patient to whom he gave a set of teeth for nothing; but in six months' time they became so perfectly *filthy*, that he advised his patient to discontinue their use. He had lately seen, however, a gentleman with a set of teeth on gutta percha, made by a dentist in Bond street (beautifully finished,) which he had worn eighteen months, and which were perfectly pure, and free from any unpleasant odor up to the present time. The difference might possibly be owing to the purer quality of the gutta percha used in the latter case. In reference to the decay produced by the use of gutta percha, spoken of by the chairman, he had seen the same sometimes produced by the use of gold clasps. He had observed, that when extensive decay took place in the mouth, the saliva was of a very glutinous character; so much so, that it might sometimes be strung out of the mouth nearly a yard long. With regard to stoppings, he had lately seen in the mouth of a lady an amalgam that he admired exceedingly. It was white, clean, and had a beautiful polish like platina, although it had been in the mouth for years. The tooth was stopped by Mr. Rogers, Jun., and he should be glad to know from that gentleman the composition of the amalgam.

Mr. Bate having handed to Mr. Thomas Rogers the name of the patient alluded to, that gentleman said, that he thought the amalgam in question must either have been Ash's, or one composed of palladium and quicksilver; but that he would endeavor to ascertain by reference to his "note book," its exact composition, and report to the society the result of his inquiry at its next meeting.

Mr. S. Cartwright, Jun., remarked, that he generally rubbed the filling on blotting paper, and found that the black oxyd was thoroughly got rid of. With regard to gutta percha, he had seen cases where it had been partially applied to the back part of plates, encircling the molars, where it seemed to answer well, and had not injured the teeth, although the plate had been worn for several years.

The Chairman said, that he formerly used silver and gold, in various proportions, combined with quicksilver, as an amalgam; but the result, as regarded the discoloration of the teeth so stopped, was not uniform, even with the same proportions of metal; and that he had at one time used, but to a very limited extent, platina combined with quicksilver for the same purpose. Since Ash's preparation had come out, however, he had almost invariably used that material. Before using this cement, he was in the habit of rubbing it in his hand, when prepared for use, with a little sal volatile, by which a large portion of black oxyd became liberated. In reference to the case referred to by Mr. Bate, he could not say whether the stopping was an amalgam of gold and silver, or platina with quicksilver, or Ash's preparation.

Mr. Spence Bate said, he had often seen Ash's stopping in the mouth, but it did not resemble the preparation in the case to which he had alluded. He might mention, before he sat down, in reference to the gutta percha stopping recently recommended to the profession by Mr. Jacob, that, five years ago, he employed gutta percha, combined with plaster of paris, as a temporary stopping, intending to stop the tooth afterwards with gold; but this had answered so well, that up to the present time the patient would not have it removed. He was inclined, from his experience in this case, to think well of such a stopping as Mr. Jacob's in lateral cavities, but would not think of employing it on the grinding surface of a tooth.

Mr. Cartwright, Jun., said, the success or non-success of gutta percha depended very much upon the state of the patient's mouth.

Mr. Harrison said, he had found bone become very hard under wear in some mouths, lasting for many years, and in some cases even retaining a very good color, although these cases were, unfortunately, the *exception* and not the *rule*, with regard to that material. He had by him a substantial set of natural teeth on bone (walrus,) which was worn for nine years by a personal friend. It had assumed a yellowish-brown appearance, was exceedingly hard, and as highly polished as if it had just come out of the workman's hands, and had not been acted upon in any way injuriously by the secretions of the mouth. He had a patient who had worn a set of artificial teeth for eighteen years. In the lower jaw they were natural teeth on bone (also walrus,) in the upper, mineral teeth on gold. The bone piece was perfectly black (being coated with a black incrustation very like a coarse black varnish,) the bone hard and sound, and if scraped presenting, a little below the surface, a brown color. The gold plate and mineral teeth were also coated with the same black material; so were the springs, which became clogged in their action, and had to be periodically renewed. He (Mr. Harrison) had originally recommended the patient to put her teeth at night in spirits of wine and water, with a view to add to their durability; but she used gin and water instead. He thought it right to mention this, although he did not suppose that the gin and water had much to do with the black incrustation spoken of, which he attributed to some peculiarity in the secretions of the mouth. In reference to hard decay in natural teeth, Mr. Harrison mentioned a characteristic anecdote of Sir Astley Cooper. Meeting him on one occasion in consultation, Sir Astley asked his opinion about a decayed tooth of his own; and, on examining it, he found it was a case of hard (or what Fox has termed "dry")

decay. He recommended that it should not be meddled with, as the tooth might last for many years in the condition in which it then was; but suggested that it should be examined from time to time. "Ah," said Sir Astley, patting him good-naturedly on the back, "I only did it to try you; Fox gave me the same opinion thirty years ago." Sir Astley had had the tooth in that state *thirty* years, and he (Mr. Harrison) believed, carried it to his grave. With regard to amalgams, one great point in using them was, to take especial care to clean the cavity very thoroughly, and make it perfectly dry before introducing them. With this precaution they would often last in the mouth for years without materially discoloring the teeth. When he first entered upon practice he was in the habit of using the amalgam made of precipitated silver and quicksilver, that being the amalgam then in ordinary use. Not being satisfied with this, he tried various other amalgams, and among them one composed of pure gold and quicksilver. This, he found, on the whole, the best, for, although it would discolor on the surface exposed to the action of the saliva, it did not *generally* discolor the tooth, when it could be introduced with the precautions before mentioned. He bore in mind a case at that moment in which he had stopped the two central incisors from behind, eighteen or nineteen years ago, in which there was no discoloration of the *teeth* apparent even now, although the amalgam on its surface next the palate looked black. Since the introduction of Ash's stopping he had occasionally used it, as also those of Lemale and Taylor, and he found that all these stoppings, if washed in pure water, when prepared for putting into the mouth (which was his custom) parted with a considerable quantity of black oxyd, which he attributed to the quantity of tin they contained. He had not submitted these stoppings to the action of sal volatile, and could not, therefore, say whether its use was preferable to that of water.

The chairman said that water would remove some of the black oxyd, spirit still more, but spirits of ammonia, or sal volatile, more than either water or spirit.

A vote of thanks having been passed to the author—

Mr. Duff, in replying, said in reference to what had fallen from Mr. Bate respecting the durability of the bone of the hippopotamus tusk, that he believed the hippopotamus tusk, as furnished now, was very different from what it was twenty-five years ago, as it seemed as if all the oily and fatty matter had been taken out by exposure to the burning suns of Africa. When the tusks were formerly supplied, some gelatine was attached to the hollow part; and he had heard that the trick was now practiced by those who received the teeth deficient in this material, of coating the hollow part with a gelatinous substance to imitate the natural gelatine.

Simon Deutz, Esq., Amsterdam, and J. B. Lindsey, Esq., Dover, were elected members of the society.

A paper by Mr. Sercombe "On a Method of taking Impressions of cases of Cleft Palate" was announced for the next meeting.

The society then adjourned.—*British Jour. Den. Science.*

